# Considerations for initial therapy in the treatment of acute heart failure

**DOI:** 10.1186/s13054-015-1114-3

**Published:** 2015-11-10

**Authors:** William F. Peacock, Chad M. Cannon, Adam J. Singer, Brian C. Hiestand

**Affiliations:** Baylor College of Medicine, 1504 Taub Loop, Houston, TX 77030 USA; Department of Emergency Medicine, The University of Kansas Hospital, 3901 Rainbow Blvd, MS1910, Kansas City, KS 66160 USA; Department of Emergency Medicine, Stony Brook University, HSC-L4-080, Stony Brook, NY 11794 USA; Wake Forest School of Medicine, Medical Center Blvd, Winston-Salem, NC 27157 USA

## Abstract

The diagnosis of patients presenting to the emergency department with acute heart failure (AHF) is challenging due to the similarity of AHF symptoms to other conditions such as chronic obstructive pulmonary disease and pneumonia. Additionally, because AHF is most common in an older population, the presentation of coexistent pathologies further increases the challenge of making an accurate diagnosis and selecting the most appropriate treatment. Delays in the diagnosis and treatment of AHF can result in worse outcomes and higher healthcare costs. Rapid initiation of treatment is thus necessary for optimal disease management. Early treatment decisions for patients with AHF can be guided by risk-stratification models based on initial clinical data, including blood pressure, levels of troponin, blood urea nitrogen, serum creatinine, B-type natriuretic peptide, and ultrasound. In this review, we discuss methods for differentiating high-risk and low-risk patients and provide guidance on how treatment decisions can be informed by risk-level assessment. Through the use of these approaches, emergency physicians can play an important role in improving patient management, preventing unnecessary hospitalizations, and lowering healthcare costs. This review differs from others published recently on the topic of treating AHF by providing a detailed examination of the clinical utility of diagnostic tools for the differentiation of dyspneic patients such as bedside ultrasound, hemodynamic changes, and interrogation of implantable cardiac devices. In addition, our clinical guidance on considerations for initial pharmacologic therapy in the undifferentiated patient is provided. It is crucial for emergency physicians to achieve an early diagnosis of AHF and initiate therapy in order to reduce morbidity, mortality, and healthcare costs.

## Introduction

Acute heart failure (AHF) is associated with high morbidity and mortality in patients presenting to the emergency department (ED). In the United States, AHF results in 676,000 annual ED visits, with over 80 % of patients requiring hospitalization [1, 2]. Hospitalization for AHF is associated with high risk for poor outcomes; more than one-third of patients die or require rehospitalization within 90 days of discharge [3]. Heart failure (HF) is also associated with substantial costs. Total estimated HF expenditures in the United States are over $39 billion/year, and by 2030 costs are projected to increase to $70 billion [4, 5]. About 68 % of HF-related costs are attributable to direct medical costs, and 80 % are related to hospitalization [5].

Accurate diagnosis of AHF is often challenging because signs and symptoms are nonspecific [6, 7]. ED physicians can use their clinical judgment to accurately diagnose AHF in about 75 % of patients [8]. Even after considering laboratory and radiographic test results, ~12 % of patients presenting to an urgent or emergency care environment with dyspnea are misdiagnosed [9]. Additionally, AHF severity is often underappreciated. A significant subset of patients with end-stage HF may be in occult shock, but are clinically indistinguishable from patients with less severe decompensation [10]. To facilitate rapid diagnosis of AHF, other tools (e.g., biomarkers, ultrasound) should be combined with clinical judgment. When B-type natriuretic peptide (BNP) or N-terminal pro-BNP levels are used in conjunction with clinical judgment, the accuracy of diagnosing AHF improves from ~75 % to ~80 % [8, 11]. However, delaying treatment initiation until comprehensive diagnostic testing is completed worsens clinical prognosis. For example, with each 4-hour delay in initiation of intravenous diuretics, the risk of in-hospital death increases for patients with BNP levels >865 pg/ml [12]. Delays in treatment may also result in increased ED and overall hospital length of stay (LOS), which can increase costs [12, 13]. Although many hospitals have developed AHF care pathways to improve patient management, there is still a lack of evidence-based guidelines for the care of undifferentiated patients with possible AHF.

This review details the importance of early diagnosis in patients presenting to the ED with AHF and discusses early treatment options for individuals both with and without a clear diagnosis. To help guide treatment decisions, methods for early risk stratification and patient differentiation are discussed. A novel discussion of diagnosis and initial treatment considerations is provided for undifferentiated patients with dyspnea.

## Review

### Early diagnosis of AHF

The diagnosis of AHF in the ED is challenging due to similarities between symptoms of AHF and other conditions such as chronic obstructive pulmonary disease (COPD), pneumonia, physical deconditioning, and sepsis [14]. In addition, the diagnostic tools available in many EDs are limited [15]. An initial physical examination is insensitive for detecting AHF [7]; electrocardiograms and chest X-ray scans can be nondiagnostic; and obtaining a full medical history can be challenging because patients presenting to the ED with suspected AHF may have an impaired ability to communicate due to dyspnea and overall poor health. These issues can result in uncertainty about the correct diagnosis and lead to delayed or inappropriate treatment, such as administration of fluids and/or antibiotics in patients initially suspected of having pneumonia, or administration of beta-agonists to patients initially suspected of having a COPD exacerbation [16]. Conversely, administering diuretics to patients with dyspnea not due to HF can worsen outcomes [17]. Accurate early diagnosis can also improve resource management by avoiding unnecessary admissions, freeing beds for other patients, and lowering costs [18].

#### Effect of treatment delays on AHF outcomes

**Fig. 1:**
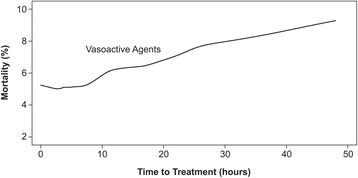
Mortality by time to initial administration of vasoactive agents. Adapted with permission from [13] © 2009 Wiley & Sons

Delayed AHF diagnosis can worsen patient outcomes by increasing the time until initiation of therapy. Such delays are associated with increased morbidity and mortality [12, 13, 19]. In an analysis of data from the Acute Decompensated Heart Failure National Registry (ADHERE), earlier initiation of intravenous vasoactive therapy with nitroglycerin, nitroprusside, dobutamine, nesiritide, dopamine, or milrinone was associated with improved outcomes in patients hospitalized with AHF [13]. On average, patients in the early-treatment group received vasoactive agents 1.7 hours after hospitalization, compared with 14.7 hours after hospitalization in the late-treatment group. If treatment was delayed by >6 hours, the adjusted odds of death increased by 6.8 % for each 6-hour delay (Fig. 1) [13]. Early initiation of vasoactive treatment was also associated with shorter length of overall, ED, in-hospital, and ICU stays.

#### Tools to aid early diagnosis of AHF

A thorough medical history complemented by vital sign readings and a physical examination can assist in determining whether a patient has AHF. However, consideration of other clinical factors can improve prognostic assessments. Laboratory tests, including complete blood count, urinalysis, serum electrolytes, blood urea nitrogen (BUN), creatinine, glucose, and natriuretic peptide and cardiac troponin levels, should be conducted. Electrocardiogram findings can narrow the differential diagnosis and detect precipitants of decompensation, such as ischemia, arrhythmias, hyperkalemia, and junctional bradycardia caused by digoxin toxicity. Comparisons with prior electrocardiograms are useful, and a truly normal reading suggests an alternative diagnosis [20]. Bedside ultrasound can help reach an accurate diagnosis. A systematic review of seven studies found that the sensitivity of ultrasound using bilateral B-lines to diagnose acute cardiogenic pulmonary edema is 94.1 % (95 % confidence interval (CI): 81.3–98.3 %) and specificity is 92.4 % (95 % CI: 84.2–96.4 %) [21]. Bedside chest X-ray scans can also provide prognostic value (Table 1) [22, 23]. However, while pulmonary venous congestion, interstitial edema, alveolar edema, and cardiomegaly increase the likelihood of AHF, their absence does not exclude AHF diagnosis [7]. Data from the ADHERE registry revealed that nearly 19 % of hospitalized patients with AHF had no signs of pulmonary congestion on chest radiography [24].

**Table 1:** Incidence of various radiologic findings in patients with AHF [22]

Finding	Incidence in patients with AHF (%)
Dilated upper lobe vessels	81
Cardiomegaly	72
Interstitial edema	72
Enlarged pulmonary artery	67
Pleural effusion	47
Alveolar edema	33
Prominent superior vena cava	23
Kerley B lines	12

*AHF* acute heart failure

#### Institutional barriers to early diagnosis of AHF

ED operations and organizational structures can delay therapy because care is focused on rapid management of other acute conditions, such as septic shock or myocardial infarction, rather than recognition and treatment of more subtle and complicated conditions such as AHF [25]. Not all hospitals have facilities to rapidly test for biomarkers. Approximately 25 % of surveyed cardiologists desired use of natriuretic peptide levels for diagnosing AHF, but lacked access to testing facilities [26]. Furthermore, there are no high-quality evidence-based guideline recommendations for the initial pharmacologic treatment of AHF. As a result, there is a lack of standardized treatment strategies [27].

Several other factors create therapeutic challenges, even when AHF diagnosis is probable. Patients with AHF tend to be older or in distress, so they are often unable to recall or communicate the medications they are taking. Because furosemide dosing should ideally be personalized to each patient’s recent baseline dose, incomplete knowledge of the patient’s recent medication regimen can result in inaccurate and ineffective therapy. Further, delays can arise while the ED reconciles information with the patient’s primary care physician, cardiologist, or pharmacy, which are frequently unreachable during nonbusiness hours. Of particular concern in EDs, the hand-off of patients during shift changes or transfer between units may also impede therapy.

### AHF risk stratification

Patients with AHF are a heterogeneous group, often with comorbidities requiring different levels of treatment. The use of risk-stratification tools can improve treatment decisions. For example, early identification of low-risk patients who are candidates for discharge or treatment in an observation unit can prevent unnecessary hospital admissions and reduce costs [28, 29]. Patients at higher risk of adverse outcomes include those with a history of hospitalizations for AHF, markedly elevated BNP (>1000 pg/ml) [30], or sodium concentration <136 mmol/l [3]. Other parameters have been stratified by dichotomous cutoff points that, if exceeded, are associated with increased risk of short-term death, including BUN ≥43 mg/dl, creatinine ≥2.75 mg/dl, systolic blood pressure (BP) <115 mmHg [31], or troponin level above the 99th percentile [32]. If these parameters are exceeded, patients may be candidates for aggressive treatment in an ICU [15]. Risk-stratification models can help guide treatment decisions, but their implementation is limited [1, 33]. In the most robust study to date, in-hospital mortality was predicted in 65,275 patients with AHF who were differentiated into risk groups based on BUN, systolic BP, and creatinine [31]. In-hospital mortality was significantly greater in high-risk (23.6 %) versus low-risk (1.8 %) patients (odds ratio (OR): 12.9; 95 % CI: 10.4–15.9; *P* < 0.001); the effect of elevated BUN, low systolic BP, and high creatinine levels on in-hospital mortality has been clearly established [31]. However, this model has limited generalizability because it was derived from retrospective data; only 39 predictor variables were evaluated, and inpatient mortality was the only outcome measured.

Risk-stratification models can also help identify low-risk patients. In a study of 33,533 patients hospitalized for AHF, a prediction rule was developed from 21 prognostic factors, including demographic and medical history variables and the most abnormal examination or diagnostic test values measured in the ED or on the first day of hospitalization [34]. Using this prediction rule, 17.2 % of patients had low risk for adverse inpatient outcomes, and these patients had lower rates of mortality (0.3 % versus 4.5 %) than the overall cohort. In a separate study, initial systolic BP >160 mmHg (OR: 1.8; 95 % CI: 1.15–2.7) and normal troponin I level (OR: 14.7; 95 % CI: 1.9–105) were independent predictors for identifying patients that were appropriate candidates for observation unit care [35]. Of 499 patients screened in this study, 27 % met the criteria for treatment in an observation unit.

#### Use of BP

**Fig. 2:**
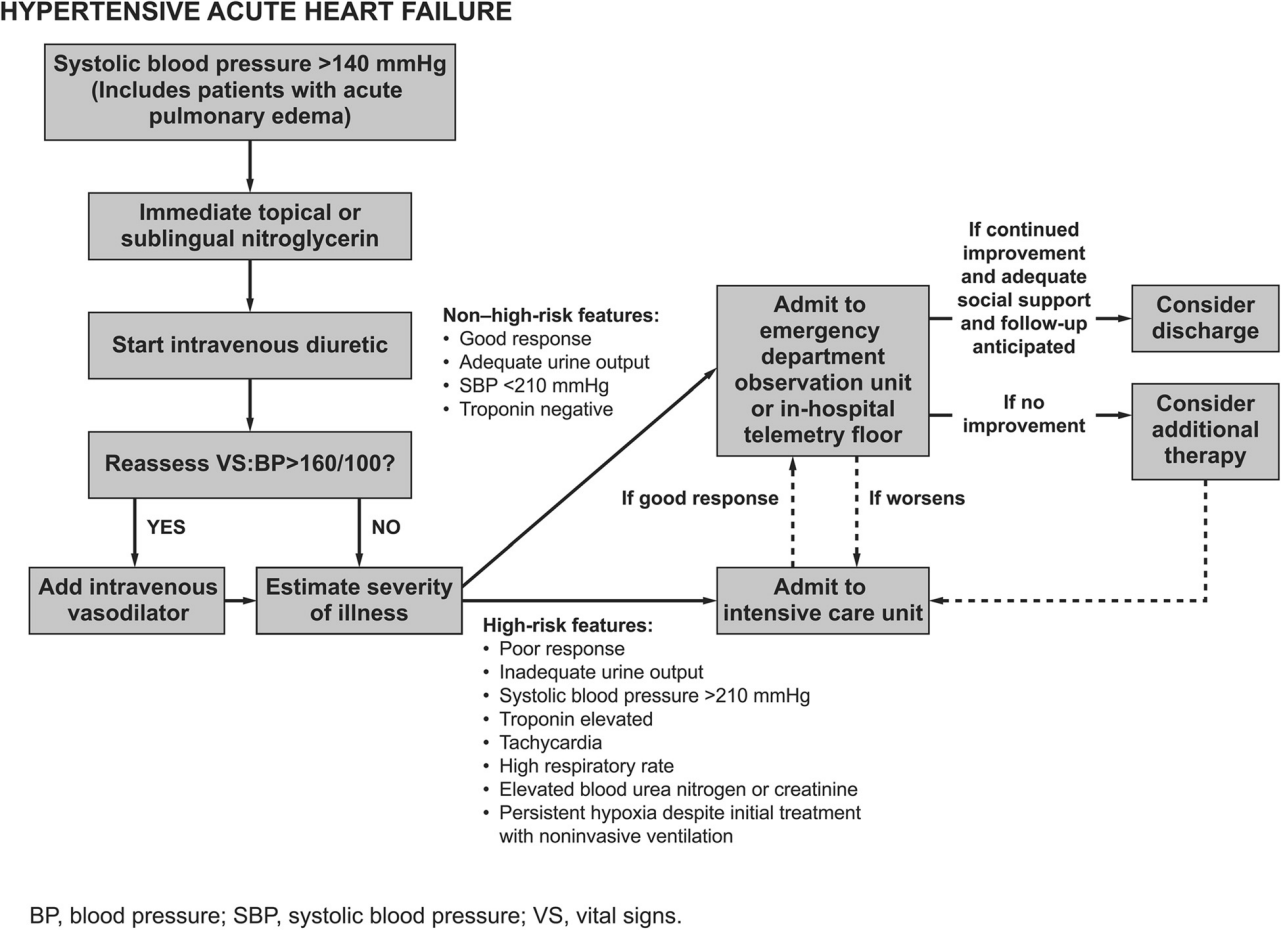
Pharmacologic algorithm for patients with hypertensive AHF. Adapted with permission from Elsevier [37] © 2008 Elsevier

BP is traditionally used to stratify patients presenting to the ED with AHF. The majority of AHF patients present within 24–48 hours of symptom onset and have systolic BP >140 mmHg [36]. This population is more likely to have severe symptoms, and acute pulmonary edema is more common than peripheral edema. With appropriate treatment, however, individuals with hypertension at presentation have lower in-hospital mortality, 60- to 90-day mortality, and a shorter LOS than nonhypertensive patients [3]. Elevated systolic BP (>160 mmHg) and normal troponin levels can define a population of low-risk patients who would benefit from care in an observation unit [35]. Treatment of these patients should focus on aggressively controlling BP and minimizing diuretic use; treatment decisions can be guided according to Fig. 2 [37]. In these patients, high-dose nitrates and low-dose diuretics may provide more consistent clinical improvement than low-dose nitrates and high-dose diuretics [38].

**Fig. 3:**
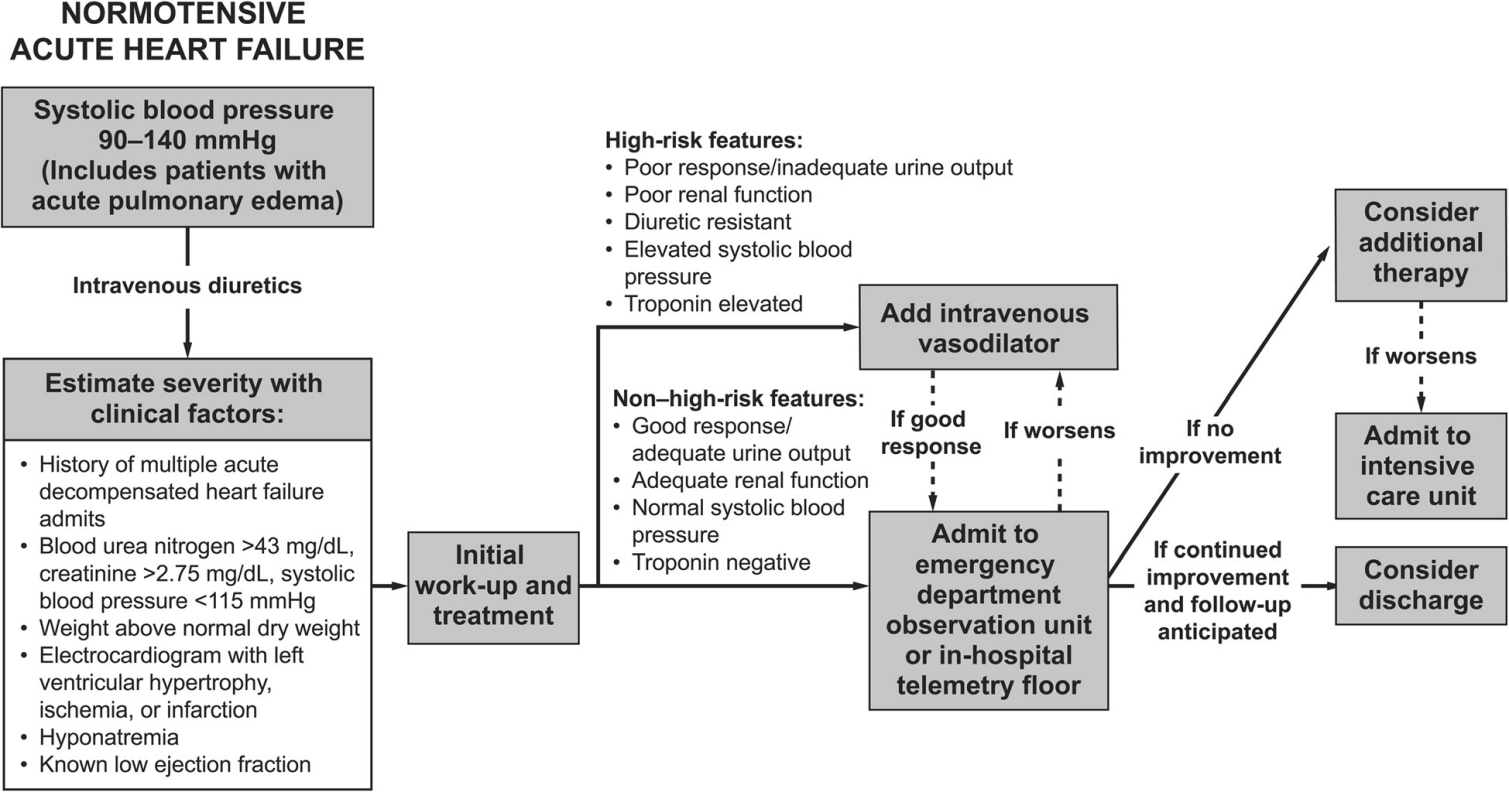
Pharmacologic algorithm for patients with normotensive AHF. Adapted with permission from Elsevier [37] © 2008 Elsevier

Approximately 35 % of patients presenting to the ED with AHF are normotensive [37]. These patients tend to be younger, have a reduced ejection fraction, have a history of coronary artery disease, and often experience mild, subacute worsening of symptoms over days to weeks before presentation. Treatment of normotensive patients should focus on aggressive diuresis to relieve congestion and reduce body weight and peripheral edema; management decisions can be aided according to Fig. 3 [37]. These patients should be monitored closely after initial therapy to ensure that their BP does not decrease beyond levels necessary for adequate perfusion.

**Fig. 4:**
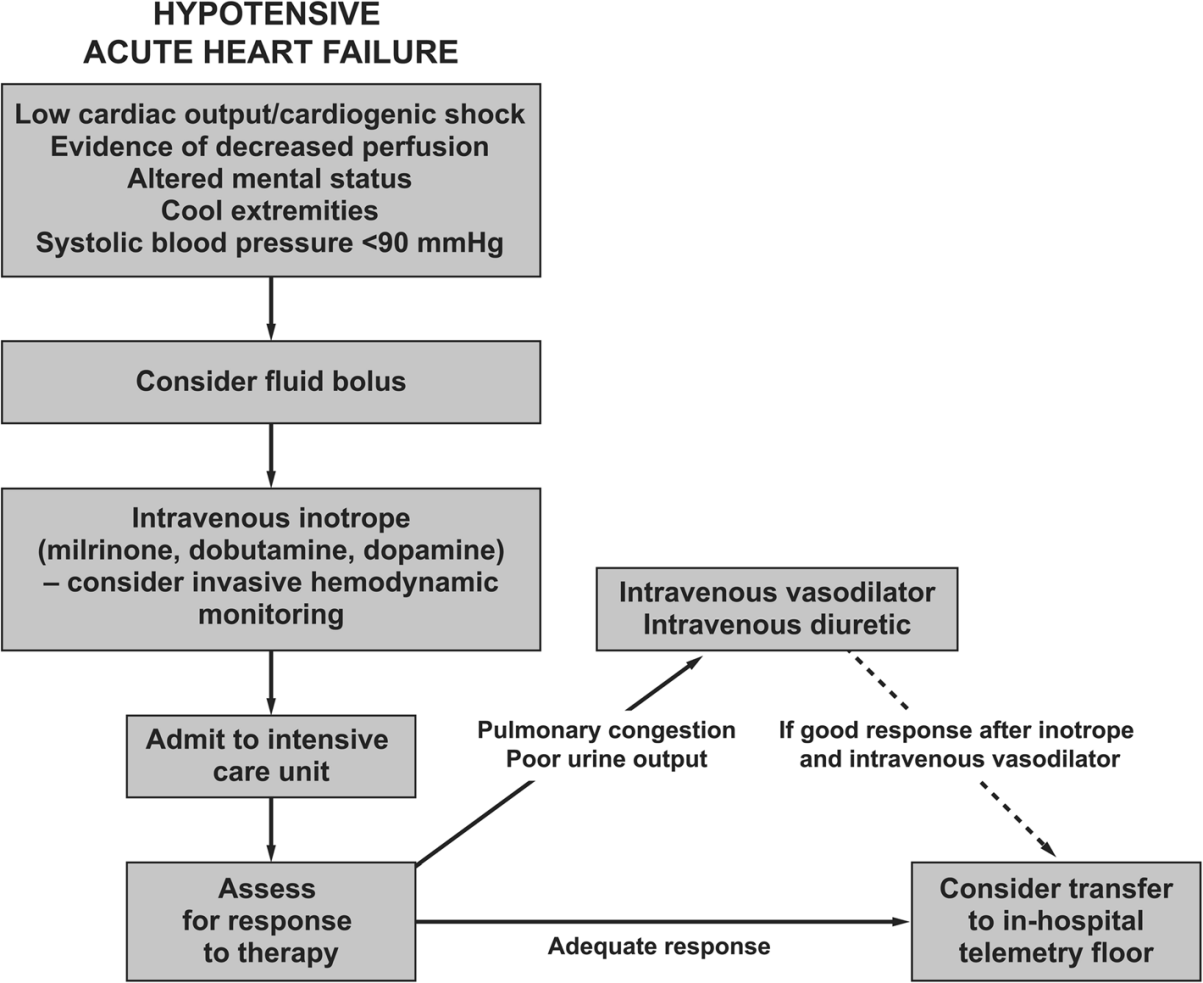
Pharmacologic algorithm for patients with hypotensive AHF. Adapted with permission from Elsevier [37] © 2008 Elsevier

Hypotensive patients (systolic BP <90 mmHg) with AHF are rare, accounting for <5 % of ED presentations, and they usually require immediate treatment due to their unstable condition [39]. Care should focus on improving hypoperfusion, not just raising BP; decisions can be guided according to Fig. 4 [37]. Owing to the severity of their condition, hypotensive patients have worse in-hospital mortality rates and require intensive care more often than other patients with AHF [40, 41].

#### Use of biomarkers

American College of Cardiology Foundation/American Heart Association (ACCF/AHA) guidelines state that BNP measurement is useful in the setting of an uncertain AHF diagnosis and for establishing prognosis or disease severity (Level of Evidence: A) [27]. The diagnostic utility of BNP in the ED was studied in the Rapid Emergency Department Heart Failure Outpatient Trial (REDHOT), in which physicians were blinded to BNP test results in patients with dyspnea [42]. BNP levels did not differ significantly between patients admitted to, and those discharged from, the ED, and physicians’ intention to admit or discharge did not affect 90-day outcomes. However, BNP levels were strongly predictive of 90-day outcomes. A substantial proportion (66 %) of patients hospitalized for AHF had low BNP levels (<200 pg/ml) yet were perceived as being moderately or severely impaired according to their New York Heart Association classification. As expected, patients with BNP levels >200 pg/ml had a higher 90-day combined event rate (HF-related ED and hospital admissions or mortality) than those with BNP levels <200 pg/ml (29 % versus 9 %; *P* = 0.006). These findings suggest there is a disconnect between the perceived severity of AHF by ED physicians and the severity defined by BNP levels.

In an analysis of ADHERE data, elevated BNP levels at admission were a significant predictor of in-hospital mortality [43]. The lowest quartile of patients had BNP levels <430 pg/ml, indicating a sizeable population that may have lower risk and not require inpatient therapy. Similar findings were observed in patients presenting to the ED with dyspnea [44]. Patients with BNP <230 pg/ml had a lower incidence of subsequent ED visits, hospitalization, or HF death at 6 months than those with levels >480 pg/ml (2.5 % versus 51 %).

**Fig. 5:**
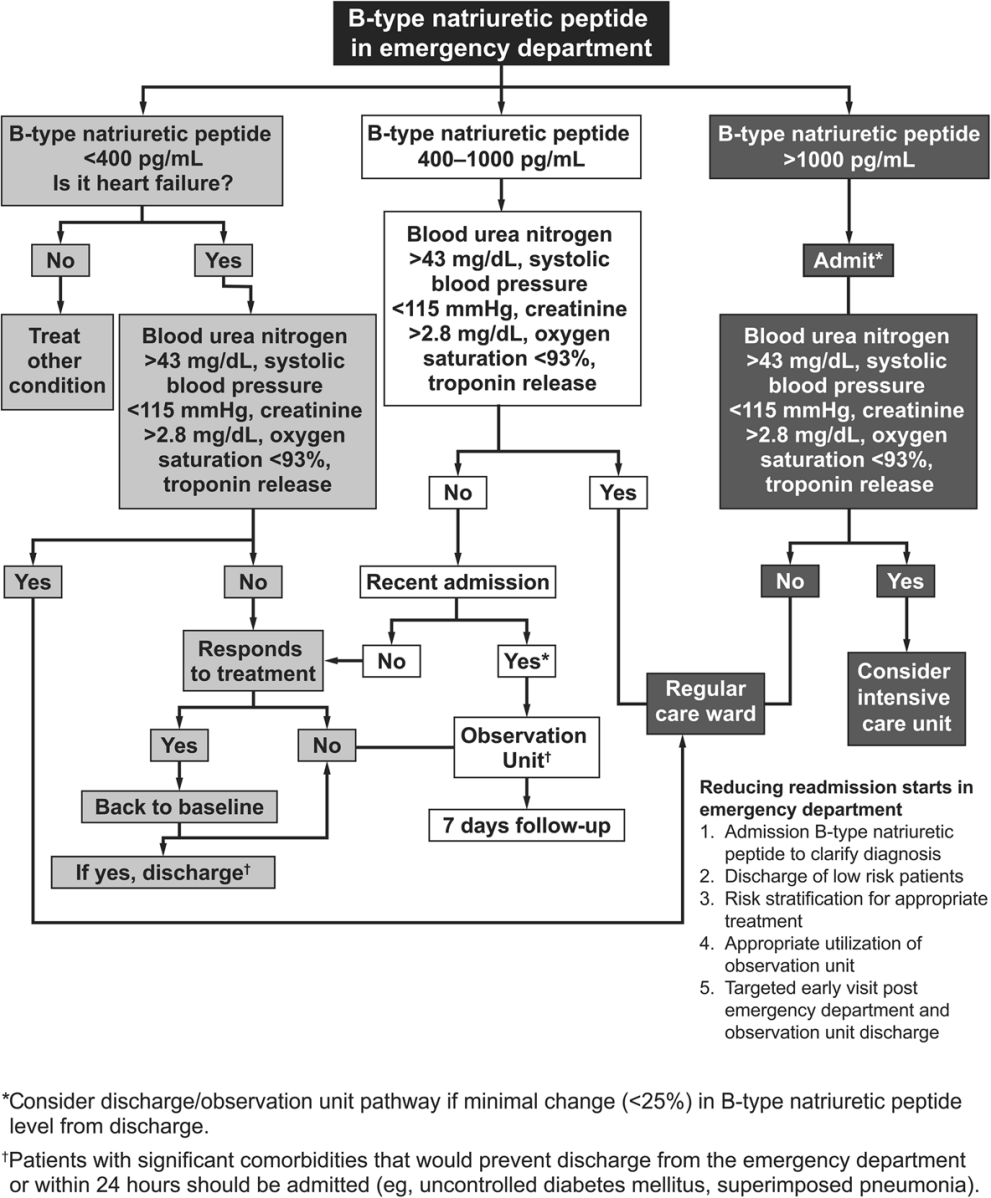
Method for identifying lower-risk patients with AHF in the ED. Adapted with permission from Elsevier [45] © 2012 Elsevier

Another European study assessed the correlation between BNP testing and resource utilization in patients presenting to the ED with dyspnea [18]. BNP testing was associated with lower hospitalization rates (75 % versus 85 %; *P* = 0.008), shorter median time to discharge (8.0 days versus 11.0 days; *P* = 0.001), and decreased ICU utilization (15 % versus 24 %; *P* = 0.01) compared with patients who did not undergo BNP testing. When used in combination with other clinical information, BNP levels can help determine whether hospitalization is necessary; Fig. 5 presents an approach for identifying lower-risk patients who may be considered for discharge or admission to an observation unit [45].

Cardiac troponin is another biomarker that provides prognostic information [32]. Patients in the ADHERE registry with elevated troponin levels were more likely to require coronary bypass grafts, balloon pumps, and mechanical ventilation. These patients also required more aggressive treatment than individuals with a negative troponin result, with more patients receiving ICU admission (37 % versus 16 %; *P* < 0.001) and having a longer LOS once admitted to intensive care (2.9 days versus 2.3 days; *P* < 0.001). A positive troponin assessment was also associated with higher in-hospital mortality than negative troponin assessment (8.0 % versus 2.7 %; *P* < 0.001).

While testing for single biomarkers is helpful, combined biomarker testing can provide additive prognostic information [30]. Patients who were positive for troponin and had BNP ≥840 pg/ml had higher in-hospital mortality rates than patients negative for troponin with BNP <840 pg/ml (10.2 % versus 2.2 %; *P* < 0.0001). These higher-risk patients were also more likely to be admitted to an ICU (32.6 % versus 14.1 %; *P* < 0.0001) and required longer LOS (5.4 days versus 4.1 days; *P* < 0.0001) [30].

#### Other factors and future considerations

Renal dysfunction is an important outcome predictor in AHF. Chronic kidney disease or worsening renal function during hospitalization, usually defined as an increase in serum creatinine of ≥0.3 mg/dl, is associated with increased short-term mortality [46]. A similar association was reported in a large-scale, randomized, controlled AHF trial in which worsening renal function was defined as an increase in plasma cystatin C of ≥0.3 mg/l [47]. Finally, patients with BUN >30 mg/dl generally require hospitalization, rather than observation unit care [48].

Changes in AHF symptoms may be prognostic for adverse outcomes. After treatment with standard therapy, dyspnea in the sitting position resolves rapidly, but similar improvements are not observed while lying flat [49]. These findings suggest an incomplete response to treatment and demonstrate the importance of monitoring both dyspnea and orthopnea [50].

### Early management of patients with undifferentiated dyspnea

Dyspnea is the primary symptom of AHF upon presentation to the ED. However, dyspnea is also common in patients with other conditions (e.g., COPD and pneumonia). Diagnosis of AHF is further complicated because COPD often occurs concomitantly with AHF [51].

**Fig. 6:**
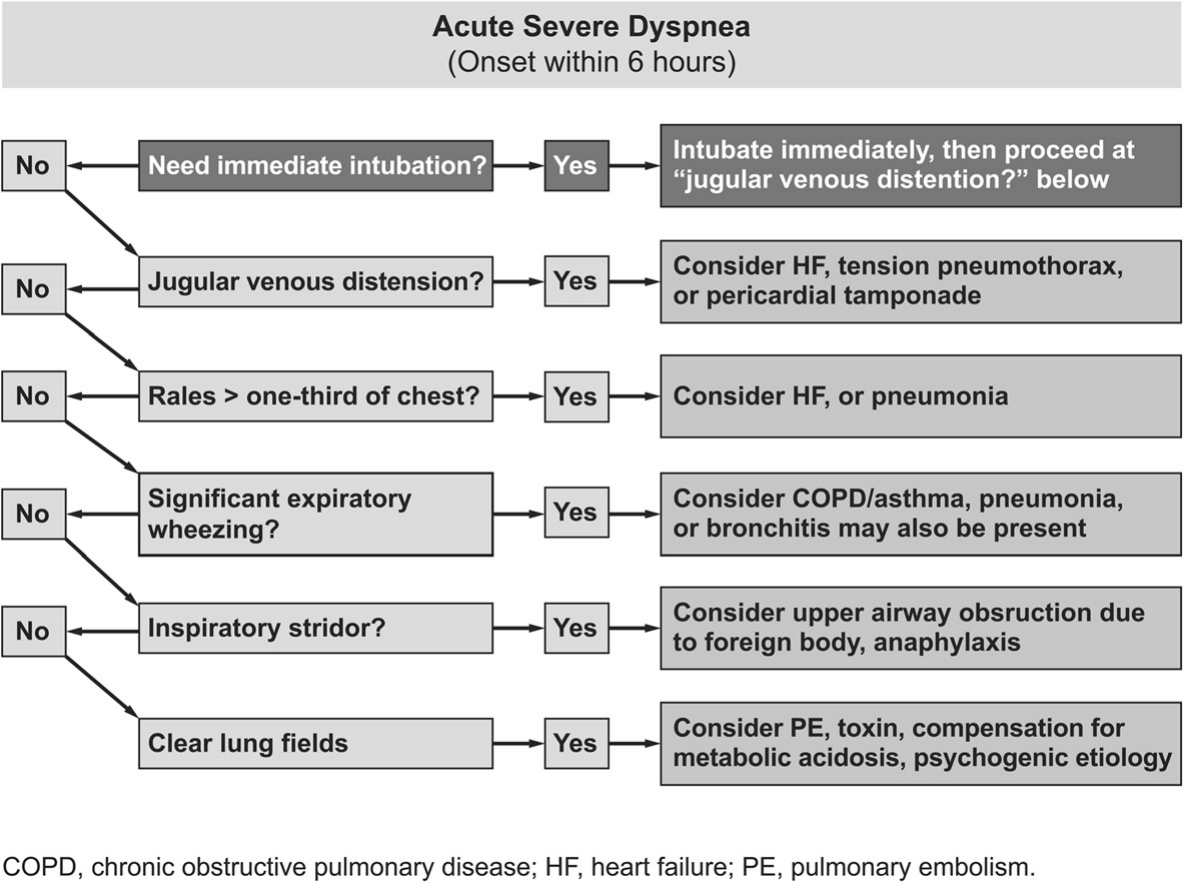
Strategy for the early differentiation of a patient with acute dyspnea. Reprinted from [53] © 2014 JAYPEE BROTHERS MEDICAL PUBLISHERS(P)LTD., New Delhi, India

To differentiate patients with dyspnea, medical history, vital signs, radiographs, and laboratory results should be considered. Additionally, echocardiography can be a useful diagnostic tool in differentiating patients with dyspnea and inconclusive BNP levels [52]. Hyperthermia or hypothermia may suggest sepsis or thyroid disease. While tachycardia is an indicator of decompensated HF, bradycardia is indicative of hyperkalemia, digoxin toxicity, beta-blocker toxicity, or atrioventricular block. Hypotension could signal severe sepsis, cardiogenic shock, cardiac tamponade, tension pneumothorax, or pulmonary embolism. A strategy for early differentiation of dyspnea is presented in Fig. 6 [53].

Patients with risk factors for both AHF and COPD and borderline BNP (100–500 pg/ml) are particularly difficult to treat. For these patients, therapy could begin immediately with agents that do not harm either condition but may provide benefit when used in the correct subset. Table 2 presents considerations for common medications used to treat dyspnea and risks associated with these medications in nonindicated patients. Of note, use of clarithromycin increases cardiovascular risk in patients with COPD and pneumonia [54].

**Table 2:** Considerations for common therapeutic agents used in the treatment of dyspnea

Agent	AHF	COPD	Pneumonia
Vasodilator	+	**○**	**○**
Inotrope	+^a^	–	–
Diuretic	+	–	–
Bronchodilator	–	++	+
Corticosteroid	**○**	++	–
Antibiotic (macrolides)	–	–	++
Noninvasive ventilation	++	++	++

^a^Risk increases in ischemic cardiomyopathy

*AHF* acute heart failure, *COPD* chronic obstructive pulmonary disease, + generally indicated, ++ strongly indicated, **○** no associated risk but not indicated, − associated risk

Noninvasive ventilation is the most well studied nonsurgical intervention that reduces mortality in critically ill patients [55]. For individuals with AHF and pulmonary edema (and most severely dyspneic patients regardless of their exact diagnosis), noninvasive ventilation is effective for early management and is a lifesaving tool in most conditions [56]. In hemodynamically stable patients with severe respiratory distress, noninvasive ventilation together with a vasodilator, a bronchodilator, and steroids may be initiated until diagnosis is clarified. Noninvasive intermittent positive-pressure ventilation can reduce the need for intubation; decrease the work of breathing; increase functional residual capacity; improve gas exchange; improve hemodynamics by reducing preload and afterload, which enhances left ventricular performance; and decrease mortality in selected patients [57]. However, noninvasive ventilation can cause discomfort and facial skin necrosis, increases risk of aspiration, and reduces venous return to the heart. Gray et al. [58] reported that while noninvasive intermittent positive-pressure ventilation did not improve mortality compared with standard oxygen treatment, it rapidly improved metabolic parameters and dyspnea. In a small study of patients with dyspnea and hypoxemia following noninvasive ventilation, therapy with high-flow nasal oxygen resulted in clinical and gasometric improvement [59].

In a pilot study, noninvasive measurement of hemodynamic changes in thoracic fluid content via bioreactance upon movement from a seated to a supine position helped differentiate AHF from COPD and asthma [60]. Patients with AHF had higher baseline thoracic fluid content and lower cardiac index responses upon postural changes.

**Fig. 7:**
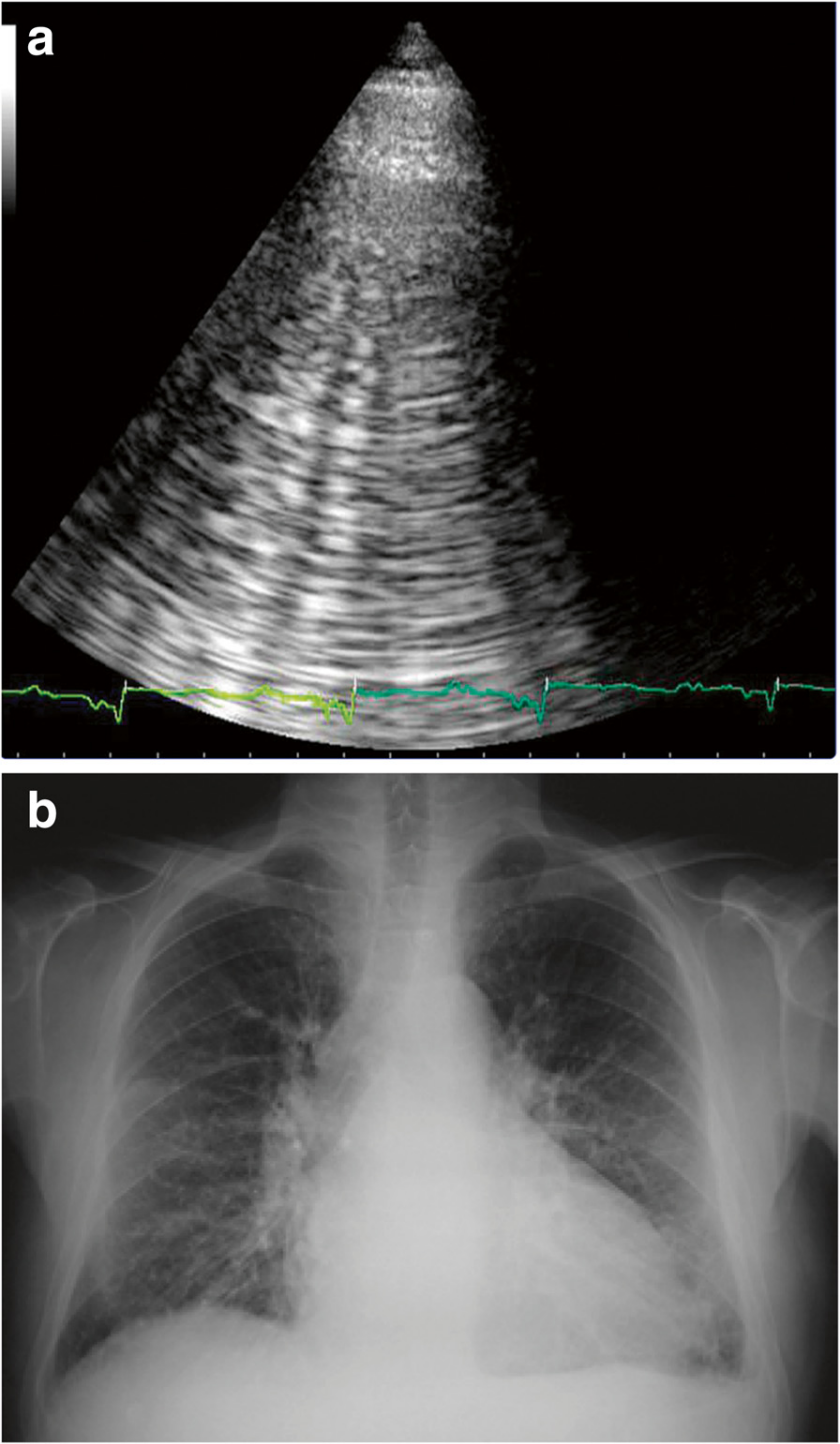
Images of B-lines from **a** lung ultrasound and **b** chest X-ray scan suggestive of AHF diagnosis. Reprinted from [23, 71]

Bedside ultrasound is another useful tool for differentiation of dyspnea. Numerous bilateral B-lines on lung ultrasound suggest AHF (Fig. 7), while unilateral B-lines suggest pneumonia. In a single-center study of 90 consecutive patients, evaluation with lung–cardiac–inferior vena cava-integrated ultrasound rapidly differentiated AHF from COPD with 94.3 % sensitivity, 91.9 % specificity, and 93.3 % accuracy [61]. In patients with borderline BNP levels, cardiac ultrasound evaluation of left ventricular end-diastolic dimension was effective for diagnosing AHF [62]. Additionally, assessing the inferior vena cava by ultrasound combined with BNP readings identified patients with AHF who were more likely to require rehospitalization [63].

Patients with AHF commonly have implantable cardiac devices that perform functions such as pacing, defibrillation, and data collection. These data can facilitate diagnosis of AHF by providing a history of atrial fibrillation burden, heart rate variability, and cardiac impedance parameters [64]. A pilot study found that ED physicians could safely and quickly interrogate implantable cardiac devices for potentially useful data [65]. Additionally, implantable cardiac devices that monitor intrathoracic impedance can predict worsening HF better than daily weight monitoring [66].

A detailed discussion of AHF treatment is beyond the scope of this review and has been covered elsewhere [39]. There is a limited evidence base for defining standard-of-care treatment. Rather, treatment decisions should be based on patient characteristics at presentation.

### The future—investigational therapies for HF

Several investigational therapies may provide new treatment options for HF. One such therapy is serelaxin, a recombinant form of human relaxin-2 that induces vasodilation. In the phase III Relaxin in Acute Heart Failure (RELAX-AHF) trial, intravenous serelaxin infusion to patients hospitalized for AHF relieved dyspnea symptoms and reduced 180-day all-cause mortality compared with placebo [67]. Another investigational therapy is ularitide, a synthetic form of urodilatin that has vasodilatory, natriuretic, and diuretic properties. In a phase II trial, ularitide lowered cardiac filling pressures and improved dyspnea compared with placebo in patients hospitalized for AHF [68]. A phase III trial of ularitide in patients hospitalized for AHF is ongoing [69]. In the near future, physicians may encounter patients with acute decompensation that are receiving LCZ696, a twice-daily oral combination of the neprilysin inhibitor sacubitril and the angiotensin receptor blocker valsartan. In a phase III trial, LCZ696 was superior to enalapril in reducing the risk of death and hospitalization for HF in patients with chronic HF with a reduced ejection fraction [70].

## Conclusion

Early diagnosis and initiation of treatment by emergency physicians is crucial for reducing morbidity, mortality, and healthcare costs of patients presenting to the ED with AHF. Risk stratification should be used to determine appropriate levels of care. When diagnosis is uncertain, initial selection of therapy is challenging, and treatment guidance can be provided by strategies for differentiation and the use of additional diagnostic tools. Patient care could be improved by development of new therapies tailored for the treatment of AHF.

## Abbreviations

ACCF: American College of Cardiology Foundation
ADHERE: Acute Decompensated Heart Failure National Registry
AHA: American Heart Association
AHF: acute heart failure
BNP: B-type natriuretic peptide
BP: blood pressure
BUN: blood urea nitrogen
CI: confidence interval
COPD: chronic obstructive pulmonary disease
ED: emergency department
HF: heart failure
LOS: length of stay
OR: odds ratio
REDHOT: Rapid Emergency Department Heart Failure Outpatient Trial
RELAX-AHF: Relaxin in Acute Heart Failure
